# Performance Metrics for Fluidic Soft Robot Rotational Actuators

**DOI:** 10.3389/frobt.2021.632835

**Published:** 2021-08-11

**Authors:** Levi Rupert, Benjamin O. Saunders, Marc D. Killpack

**Affiliations:** Robotics and Dynamics Lab, Department of Mechanical Engineering, Brigham Young University, Provo, UT, United States

**Keywords:** soft robot metrics, fluidic actuators, performance, evaluation, benchmarking, soft robotics

## Abstract

The field of soft robotics is continuing to grow as more researchers see the potential for robots that can safely interact in unmodeled, unstructured, and uncertain environments. However, in order for the design, integration, and control of soft robotic actuators to develop into a full engineering methodology, a set of metrics and standards need to be established. This paper attempts to lay the groundwork for that process by proposing six soft robot actuator metrics that can be used to evaluate and compare characteristics and performance of soft robot actuators. Data from eight different soft robot rotational actuators (five distinct designs) were used to evaluate these soft robot actuator metrics and show their utility. Additionally we provide a simple case study as an example of how these metrics can be used to evaluate soft robot actuators for a designated task. While this paper does not claim to present a comprehensive list of all possible soft robot actuator metrics, the metrics presented can 1) be used to initiate the development and comparison of soft robot actuators in an engineering framework and 2) start a broader discussion of which metrics should be standardized in future soft robot actuator research.

## 1 Introduction

Promising characteristics of soft robots include the ability to safely interact in unmodeled and unstructured areas around sensitive equipment, materials, or humans. Many of the proposed safety benefits of soft robots derive from their compliant actuation and structure, their reduced mass, and their compliant external surface. Despite the commonalities in many soft robot designs, many researchers continue to develop new types of actuators (involving novel actuation methods, geometries, materials, or combinations) without a clear way to compare their performance. These new designs include actuation methods ranging from tension cables, to Shape Memory Alloys (SMA), to fluidic actuation. Not only are there different methods used to provide “power” for the actuators but the style and shape of the many actuators differ.

Despite a variety of soft robot actuators, there is currently no systematic way for someone to determine which actuator will be most advantageous for a prescribed application or to design different actuators based on required performance specifications. This is due to the lack of a standardized method for comparing different soft robot actuators. For the design and control of soft robotic actuators to emerge as an engineering methodology, a set of metrics for comparison needs to be established. This article attempts to lay the groundwork for that process and encourage a broader discussion about engineering design and evaluation for soft robotics, specifically soft robot actuators.

The contributions of this paper are as follows:

• A brief survey of soft robot actuators and metrics
• A justification of the necessity for soft robot actuator metrics
• Development of six proposed soft robot actuator metrics and methods for how fluidic rotational actuators are evaluated using the metrics
• An application and evaluation of the metrics using data from eight different soft robot actuators (five of which use distinctly unique actuation methods, while the others include variations in material or geometry)

While not a direct contribution of this paper to the soft robotics field, we also include a case study as an example of how the metrics can be implemented to evaluate the actuators with respect to a specific task.

It is important to note that while the data and analysis of the actuators is of significance, it is not the main focus of this work. The data are used to support the development of the six proposed soft robot actuator metrics. Also of note is that while these metrics and methods are only validated for fluidic rotational soft robot actuators, we discuss methods for adapting them to other types of soft robot actuators in Section 4. The paper is organized as follows.

We first describe the state of the art with respect to soft robot actuators as well as the state of the art for soft robot actuator metrics in Section 1.1. We then define our metrics and describe how they are measured in Section 2.1. Next we develop the methods that we use in this paper to validate the metrics including a scoring method as described in Section 2.2. In Section 2.3 we describe the actuators that will be used in this work to validate the proposed metrics, as well as any actuator specific variations to the methods used to measure the metrics. Section 2.4 introduces a case study where we provide an example of using the proposed metrics to select actuators for an example task. Next, Section 3 shows the results of the tests and discusses the different metrics, how they differentiate the variety of actuators, along with an evaluation of the metrics. The details and analysis for the case study are presented in Section 3.9. Lastly, Section 4 concludes the paper, discusses the limitations of the work, and discusses potential applications and future work.

### 1.1 Related Work

Various actuator types exist in the field of soft robotics (as described by Rus and Tolley, 2015; Hines et al., 2017). These actuator types each have their advantages and disadvantages, however most of the actuators that lift significant loads use either tendon actuators or fluidic actuators (see Rus and Tolley, 2015; Best et al., 2016; Laschi et al., 2012; Sanan et al., 2011; Hannan and Walker, 2003). In this paper, we focus on fluidic actuators as they represent a prominent portion of the soft robot actuators being developed. The following section reviews the current state of art for fluidic soft robotic actuators in more detail. However, many of the metrics described in this paper could be adapted to non-fluidic actuators.

#### 1.1.1 Fluidic Soft Robot Actuators

One of the earliest fluidic actuators is the Pneumatic Muscle Actuator (PMA) or McKibbon actuators (Schulte, 1961; Daerden and Lefeber, 2002). These actuators use a cylindrical strain limiting outer layer and a cylindrical flexible internal bladder. When pressurized the PMAs naturally minimize the ratio of volume to surface area thus contracting. The relationship between pressurization and loads has been thoroughly explored and documented (Daerden and Lefeber, 2002). PMAs continue to be used in the design of new soft robotic actuators (Ohta et al., 2018) due to their use of lightweight and simple elements. PMAs are most often used in multiples of two where they are arranged in an antagonistic format so that the PMAs pull in opposite directions. It is the difference in forces/torques that then causes a net force/torque on the load.

Another subset of fluidic actuators are Fluidic Elastic Actuators (Marchese et al., 2014; Shepherd et al., 2011) which rely upon the elastic nature of the material from which they are fabricated. They often have several chambers which are flexible and deformable with strain limiting sections that help to transfer the pressure in the chambers to create forces and/or motion. They are used in a myriad of applications including grasping (Galloway et al., 2016), large body motion (Marchese et al., 2014), and even locomotion (Shepherd et al., 2011; Duggan et al., 2019).

Rotary Elastic Chambers (Rotary Elastic Chamber) (Mihajlov et al., 2006; Best et al., 2016; Hofer and D’Andrea, 2018) are able to provide rotary motion similar to traditional motor actuators but they do so without any gearing or moving seals. They have been used to show reliable control and to perform simple tasks (Best et al., 2016; Hyatt et al., 2018). Rotary Elastic Chambers are also antagonistic in nature.

Continuum actuators are another type of soft robot actuator. Many continuum actuators in the literature use tendons to cause motion or exert force, while others use fluid power (Rolf and Steil, 2012; Godage et al., 2012; Giannaccini et al., 2018; Jones and Walker, 2006; Bodily et al., 2017). Continuum actuators have no defined center of rotation and even some Fluidic Elastic Actuators are considered continuum actuators. Many continuum actuators are distinctive in their ability to bend in two degrees of freedom (DOF) with a single mechanism. Some continuum actuators have a third DOF as they can also grow in length (Giannaccini et al., 2018).

Other fluidic actuators include origami inspired artificial muscles (Li et al., 2017) which use a folding “skeleton” that is housed in a sealed “skin.” The folding skeleton provides the path for actuator motion as air is removed from the skins. Many different motions can be achieved by changing the geometry of the origami skeletons and has been shown to achieve linear as well as grasping motions. Another fluidic actuator that is able to grow and is based on the principle of eversion. Hawkes et al. (2017) developed an actuator that is made of an inverted thin membrane that can be deployed from a roll. As air is added to the roll the internal pressure causes the roll to evert which results in “growth.” The authors were able show the ability to control the direction of growth of the actuator through changing the length of material at the tip of the actuator.

While not a complete survey, the majority of the different types of fluidic soft robot actuators are represented above. In this paper we use our proposed metrics to compare different types of Fluidic Elastic Actuators, Rotary Elastic Chambers, and fluidic continuum actuators.

In Section 1.1.2, we next discuss the current state of soft robot actuator metrics.

#### 1.1.2 Actuator Metrics Related Work

The area of soft robot metrics is a new and emerging field. Mosadegh et al. (2014) developed five parameters to characterize the performance of their actuator relative to the original design from the Soft Robotics Toolkit (Holland et al., 2014). However, the focus of the paper was on a new specific type of soft robot actuator design while our paper is focused on the development of general soft robot actuator metrics. Similarly, Krause et al. (2019) developed a test bed for testing and evaluating Fluidic Elastic Actuators. However, this test bed does not appear to generalize well to any other type of soft robot actuator so that its application is limited.

Joshi and Paik (2019) present methods and experiments to characterize a Fluidic Elastic Actuator in multiple directions and loading conditions. This is a significant step in the direction of developing metrics for soft robot actuators as it starts to establish a method for characterizing and comparing soft robot actuators, albeit limited to a single type of actuator. Agarwal et al. (2016) develop some metrics for a subset of soft robot actuators by surveying the current literature of assistive wearable devices. Using designs found in the current literature they developed metrics from which they were able to base their new design. They showed that their new design was able to meet their metric objectives as compared to the surveyed literature. Their scope was also limited to a specific type of soft robot actuator, wearable assistive devices, and does not generalize well.

Morzadec et al. (2019) used soft robot finite element models to evaluate soft robot designs using a torque based fitness function. They then used an evolutionary algorithm to iterate upon the design to find an “optimal” design of a deformable leg for a locomotive robot before they built the robot. Although the metric that was used is specific to their use, this shows the potential of using a metric to aid in the design of soft robots through computer aided tools. By having a common set of metrics the tools can also become more general and useful for the soft robotics community.

Garriga-Casanovas et al. (2018) developed a method for describing Fluidic Elastic Actuator soft robotic actuators with the intention of unifying the way they are described across the literature. In it they discuss how different design parameters affect metrics such as force and deflection. In our work, we instead look directly at developing metrics that can be used for comparison across many different soft robot actuators and not at how the design of actuators affect their performance with respect to metrics. We expect that developing a solid foundation of well-defined metrics as proposed in this paper will lead to superior and more robust design tools for soft robot actuation.

## 2 Materials and Methods

We first discuss our proposed metrics and the methods used to collect data and calculate the metrics for our actuators. Next, we present the methodology that we used to evaluate these metrics. Then we describe the actuators that we used to collect data, calculate metrics, and then evaluate those metrics. Last we present an example case study of how the proposed metrics could be used to select an actuator for a given task.

### 2.1 Soft Robot Actuator Metrics and How They are Measured

We propose the following soft robot actuator metrics along with their descriptions and methods for measurement.

• Maximum Torque
• Torque-to-Mass Ratio
• Efficiency
• Parasitic Stiffness
• Variable Stiffness
• Maximum Range of Motion (range of motion)

In the results section of this work we also use the mass of each actuator as a baseline metric for evaluation of our proposed metrics.

We do not claim that this is a complete or exclusive set of metrics for all soft robot actuators, nor do we claim that the methods we use to measure them are the best for all actuators. However, we show that these metrics generalize and allow comparison between different fluidic rotational actuators. All the metrics are chosen to be agnostic to control methods or the dynamics of the systems, therefore all measurements are taken at static values after any transient dynamics have subsided. This provides the best comparison between actuators using these specific metrics. Combined metrics for actuation plus control is outside the scope of this paper, but is a significant subject for future research.

The following equipment was used to gather the data for all of the experiments:

• Motion tracking system [either infrared cameras with sub mm accuracy, or HTC Vive Trackers with sub cm accuracy for static measurements (van der Veen et al., 2019)] to accurately track the pose of the actuators’ top and base.
• Six DOF force and torque (FT) Sensor from ATI Technologies (Axia80-M20)
• Pressure control system that has embedded electronics, pressure sensors, valves, and a Real Time computer (RTPC)
• Computer to record data
• Scale for weighing actuators

All the data from the motion tracking computer and RTPC were transmitted using Robot Operating System (ROS) messages with time stamps which enabled us to time sync them for analysis. During post processing for every metric we extracted data corresponding to the same positions and ranges to be used for all calculations.

The relative angular deflections of the actuators are calculated from the pose of their top and base using the method described in Hyatt et al. (2018). Additionally each actuators’ base is mounted solidly to keep the base from moving during actuation and force measurements. We did not use a standard fixture between all the actuators due to the widely varying designs of each actuator. This meant that most actuators required their own unique fixture. The exception to this approach was that all of the Fluidic Elastic Actuators had the same fixture. The processes described are adaptable to many different types of actuators due to this fact. Standardization of the fixturing and measurement method itself seems difficult given the wide variety of actuators. However, we do expect that reporting on noise and repeatability characteristics of a measurement device and fixture is likely important for future results that use the metrics we present in this paper to be general.

#### 2.1.1 Maximum Torque

Just as it is important to know the torque limits of an electric motor or hydraulic actuator, it is crucial to know the torque limits for soft robot actuators. For fluidic rotational actuators, the two major contributions to their maximum torque is first, the actuator’s ability to convert pressure to force and second, the maximum pressure the actuator can handle without failing.

The Maximum Torque was found by controlling the actuator to its maximum operating pressure and letting the actuator come to rest. The authors then used the FT sensor to push the actuator back to its un-deflected (or neutral) configuration. The measurement was taken once the system was at rest and all transient dynamics had died out.

#### 2.1.2 Torque-To-Mass Ratio

The Torque-to-Mass Ratio compares the weight of the actuator to its maximum torque. This metric includes the weight of all the elements of the joint that are integrated into the actuator as a system. Many applications for soft actuators involve them being mounted on a mobile platform, attached on the distal end of another actuator, or some other mode where the actuator’s full mass is being actuated. Therefore for each application, the full mass that is being accelerated should be used. If it is just the distal end of the actuator being accelerated, the mass associated with that segment should be used. Similarly, if the full actuator will be actuated by another actuator then the full mass should be used. Any part of the system that can have a another device or model be substituted in its place should not be included in the mass measurement. For example, the actuators presented in this paper need pressure regulators, pumps, tubing and controlling electrical hardware to have a fully functioning actuator. Since the actuators could still work with any type of functioning pressure regulator, valves, tubing or pressure controlling electrical hardware that met the specifications, these were not included in the mass of the actuator since they did not affect the performance or function of the actuator. Only tubing that was self contained in the actuator was included in the mass measurement. Section 2.3 discusses what masses we use for our metric evaluations.

The mass of the actuators were measured using a scale, which was then combined with the previously defined Maximum Torque to calculate the Torque-to-Mass Ratio. The ratio is calculated by dividing the Maximum Torque by the mass of the actuator.

**FIGURE 1:**
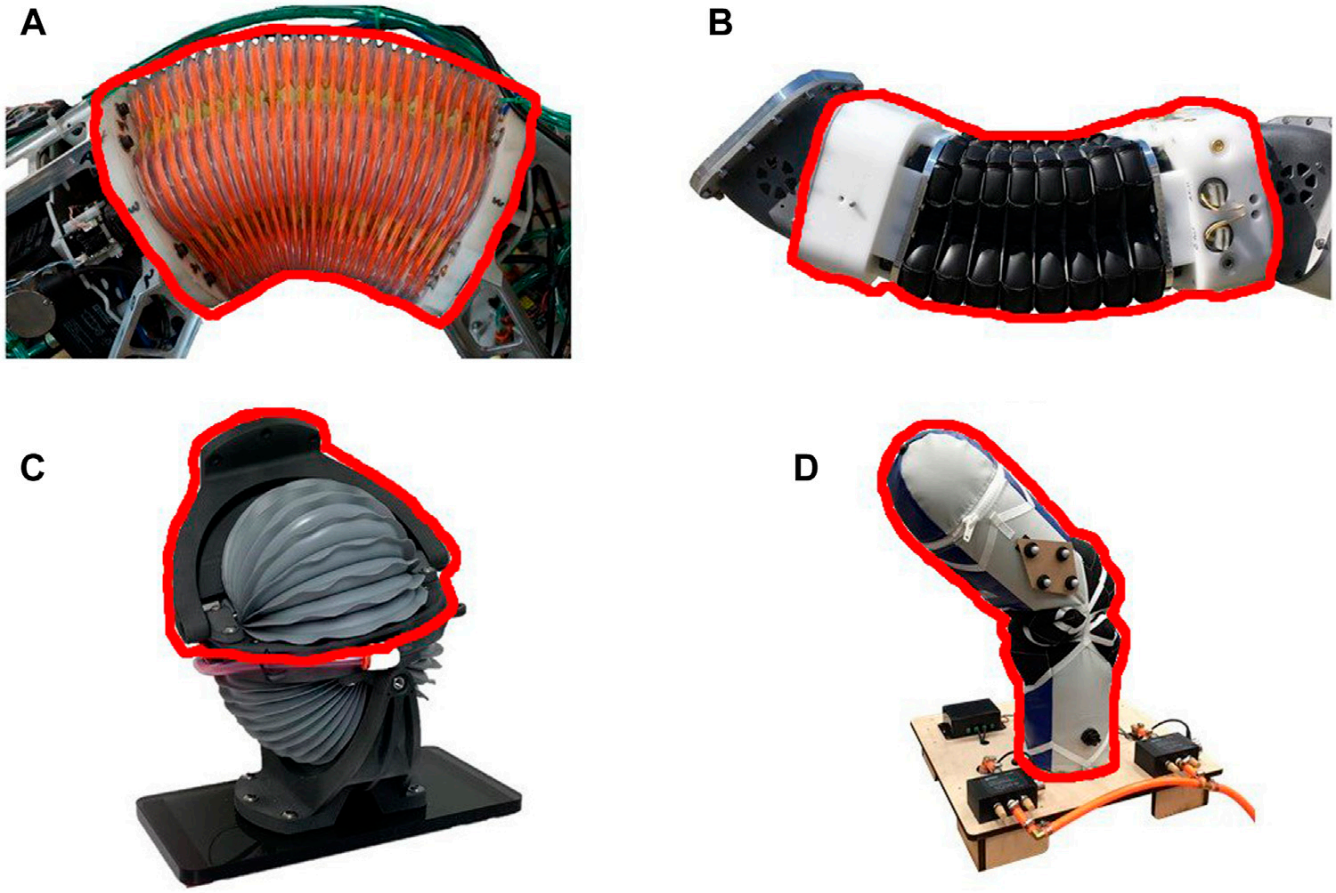
Large soft robot actuators used in this work. The highlighted sections in red are the sections used in the mass and Torque-to-Mass Ratio metrics. **(A)** Blow-molded Continuum actuator **(B)** Bead Continuum actuator **(C)** Rubberized Rotary Elastic Chamber actuator **(D)** Fabric Rotary Elastic Chamber actuator

For the actuators used in this paper the sections highlighted in red in Figure 1 are the sections used for the mass and Torque-to-Mass Ratio metrics. These sections were selected as we assume the full actuator will be accelerated and they represent the minimum required components for the actuators to be used. If any other parts were left out from the sections highlighted in red the actuators would be structurally unsound. As stated before no pumps, or pressure regulators were part of the mass measurements and only tubing that was an integrated part of the actuators was included.

#### 2.1.3 Efficiency

All motors used in traditional robots have a known efficiency. For soft robot actuators this measurement should also be used as a method to compare performance. The standard measure of efficiency is a ratio of the energy output of an actuator divided by the energy input for the actuator. For fluidic soft robot actuators we must define an equivalent energy input for the system as well as a way to measure the energy output of the system.

For pressure driven soft robot actuators, when at a static state, i.e., at equilibrium, pressure can be used as a measurement of the energy density of a system. We therefore used pressure as a measurement of the energy that we put into the system. As described in the Kinetic Theory of Gases (Clausius, 1857) the average molecular kinetic energy of a system is directly proportional to the pressure and volume.

$$E=\frac{3}{2}PV$$ (1)

Since we are interested in the change in kinetic energy as the actuators are pressurized we take the difference as follows.

$$\Delta E=E_{2}-E_{1}$$ (2)

where

$$E_{i}=\frac{3}{2}P_{i}V_{i}\;\;\;i=1,2$$ (3)

*E*_1_ is the energy of the actuator when its internal pressure is equal to that of the atmosphere, and *E*_2_ is the energy of the actuator when actuated to any other pressure. Additionally, the pressure and volume at state 2 can be expressed as a change from state 1 as follows:

$$P_{2}=P_{1}+\Delta P$$ (4)

$$V_{2}=V_{1}+\Delta V$$ (5)

By substituting in Eqs 4, 5 into Eq. 3 and substituting those into Eq. 2 the following equation for the change in energy is calculated.

$$\Delta E=\frac{3}{2}\left(\Delta PV_{1}+P_{1}\Delta V+\Delta P\Delta V\right)$$ (6)

Additionally, for our measurements, we assume that the actuators undergo negligible change in volume at each data sample as they are measured at the same angular position each time and the actuators used do not undergo large amounts of strain during pressurization. Therefore we can simplify Eq. 6 further such that

$$\Delta E=\frac{3}{2}\Delta PV_{1}$$ (7)

Eq. 6 should be used instead for soft robots that undergo significant change in volume as their internal pressure increases.

To determine efficiency, we also need to calculate the energy output of the system. The energy output of a system is usually measured by how much work is done. However, we take static measurements at the same angular position each time (the undeflected position) and since there is negligible change in volume we assume that no work is being done. We instead measure the force that is being produced by the actuator. Knowing the geometry of the actuator, we calculate the torque produced by the actuators. The units for efficiency will be *Nm*/*J* as it is the relationship between energy in the actuator to static torque produced by the actuator. Although *Nm* and *J* have the same base units ($\frac{kg\cdot m^{2}}{s^{2}}$) values greater than one are possible because it is not a direct measurement of the work in compared to the work out of the actuator. This is due to the static nature of this metric.

It is important to note that this metric neglects any of the losses that occur during the process of pressurization for the following two reasons. First, the compressor or flow losses that occur prior to the actuator are independent of the actuator design and so should not be used for making actuator comparisons. Second, this metric is a static measurement, therefore any dynamic losses (like flow losses) of the actual actuator are also neglected. Additional dynamic measures of efficiency are worth investigating in future work.

In order to calculate efficiency as previously described, the data gathering was completed using the following steps:

1. Two motion tracking frames were initialized to be in the same orientation on the bottom and top of the actuators in their un-deflected or neutral configurations.
2. The actuators were controlled to a known pressure and the actuator was allowed to come to rest.
3. The FT sensor was then used to push the actuators back to the approximately un-deflected state while recording data (i.e. pressures, pose of top and base, angular deflections, forces and torques).
4. The FT sensor was removed and the actuator was allowed to deflect under the actuation load from the pressure until the actuator came to a rest again.
5. The pressure in the actuators was than incremented and the actuator was allowed to come to rest.
6. Steps 3–5 were repeated until the actuators maximum operating pressure was reached.

For the antagonistic actuators one side was controlled to a constant low pressure while the other was incremented.

A video showing an example of how the data were taken for the Fluidic Elastic Actuators can be found here https://youtu.be/KPYLk_J_QxA.

#### 2.1.4 Parasitic Stiffness

Most soft robot actuators use elastic elements in their construction which results in an inherent spring force. When unactuated, the spring force will return the actuator to a neutral pose or natural equilibrium configuration. At positions away from neutral this stiffness reduces the torque available from the actuator and is parasitic in nature. Generally, torque loss due to parasitic stiffness increases with deflection from the neutral pose. This stiffness is a property of the material and geometry of the actuator and depending on the geometry of the actuator is independent of the pressure in the actuator. It should be measured while at an un-pressurized state. The units are those for standard rotational stiffness, *Nm*/*rad*.

Understanding the parasitic stiffness enables soft robot designers to know how their torque changes over the operational space of their actuator. Some applications may require a minimal parasitic stiffness while other applications may only require that the available torque over the operational space be higher than a given threshold. Combining this metric with the Maximum Torque metric can help estimate the torque capabilities over the whole range of motion of the actuator.

The parasitic stiffness was measured through the following process:

1. Two motion tracking frames were initialized to be in the same orientation on the bottom and top of the actuators in their un-deflected or neutral configurations.
2. The FT sensor was used to push the actuators to a known and consistent displacement while the actuators are fully vented allowing for no pressure differential to build through compression of the chambers.
3. The FT sensor was removed and actuator was allowed to come to rest at the zero configuration.
4. Steps 2 and 3 were repeated several times while recording data (pose of top and base, angular deflections, forces, and torques) in order to calculate an average parasitic stiffness.

#### 2.1.5 Variable Stiffness

As many soft robot actuators are only able to produce force or torque in a single direction, they are often combined into antagonistic actuators. Therefore, many of the antagonistic soft fludic actuators have the ability to vary their stiffness by adjusting the average pressure in their antagonistic chambers. As this average pressure is increased or decreased the stiffness also increases or decreases. Gillespie et al. (2016) and Best et al. (2020) show that this stiffness can be controlled while still controlling joint angles. This is a distinct advantage of antagonistic actuators over traditional motor actuators and therefore a metric capturing this distinction is critical.

This metric measures the ability an actuator has to vary stiffness. For this metric we report how changing the average energy in the chambers affects the stiffness of the actuators. Similar to the Efficiency metric we relate the pressure in the chamber to the potential energy in the system through the volume contained in the actuator as described in Eq. 2. The units of this metric are a rotational stiffness per average chamber pressure, *Nm*/(*rad* ∗ *J*).

The variable stiffness was measured in a method similar to the efficiency metric using the following process:

1. Two motion tracking frames were initialized to be in the same orientation on the bottom and top of the actuators in their un-deflected or neutral configurations.
2. Opposing chambers of the actuators were controlled to a known common pressure and the actuator was allowed to come to rest at the neutral configuration.
3. The FT sensor was used to push the actuators to a known and approximately consistent displacement while recording data.
4. The FT sensor was removed, the pressure in the actuators was than incremented and the actuator was allowed to come to rest again at the zero configuration.
5. Steps 3 and 4 were repeated until the actuators maximum pressure was reached.

#### 2.1.6 Maximum Range of Motion

As many servo motors or rotary sensors have a limited range of motion, for many applications it is important to know the range of motion for soft robot actuators. When designing a new soft robot actuator or selecting one for a given application the range of motion is a critical consideration. This measurement will be reported as a positive or negative range from the neutral configuration with the following format (Minimum Angle, Maximum Angle) and will be in radians (rad).

This range of motion was measured by bending each actuator to its maximum displacement. For all the antagonistic actuators, except the Blow-molded continuum actuator, this was done while at atmospheric pressure. The high parasitic stiffness of the Blow-molded continuum actuator required that a single chamber was pressurized to bend it to its limits. The Fluidic Elastic Actuator actuators range of motion were also measured while pressurized to their maximum pressure.

The range of motion metric for the Fluidic Elastic Actuator actuators used in this paper (described in Section 2.3.2) is only one-sided as they can only bend in a single direction.

### 2.2 Metric Evaluation Criteria

One of the objectives of this paper is to determine the ability of each proposed metric to quantify the performance of soft robot actuators. Each Metric Evaluation Criteria (MEC) is chosen to enable the development of general metrics that allow for effective comparison across the many different features and details that make each actuator unique. The MEC that will be used are the following:

• Task Utility
• Design Comparison
• Information

#### 2.2.1 Design Comparison

The MEC examines how well each metric allows for exploration of the trade offs that are present during design of novel soft actuators.

#### 2.2.2 Task Utility

The Task Utility MEC evaluates how the metric can inform the user about the direct utility of the actuator in terms of a task or application. Although this may seem like a trivially important metric, we feel that it must be included as an MEC. If a metric cannot compare the usefulness of an actuator with respect to a task it is not a valid metric.

#### 2.2.3 Information

This MEC explores if the metric has different information about the actuators when compared to other metrics.

Each metric will be evaluated for each MEC with a binary choice. “Yes” if the metric satisfies the MEC or “No” if it does not. It is understood by the authors that these evaluations are somewhat subjective but are based on “Engineering Judgment” as is common for many engineering design tasks that require creativity that is nonetheless grounded in math, physics, etc. (see Pahl and Beitz, 2013). In addition, future work on quantifying how well these or other metrics meet the proposed MECs is important work.

### 2.3 Actuators Used in This Work

This section describes the fluidic actuators used in this paper. The two major different types of fluidic actuators used in this work are antagonistic and non-antagonistic. Each type will be discussed in the following subsections as well as any specifics for calculating metric values for the different actuators. A video of the actuators and their actuation can be found here https://youtu.be/OT_D5RqlIi8.

A list of the different actuators in this work includes the following:

• Bellows Continuum Joint
  • Blow-molded Continuum
  • Bead Continuum
• Rotary Elastic Chamber
  • Rubberized Rotary Elastic Chamber
  • Fabric Rotary Elastic Chamber
• Fluidic Elastic Acutators
  • Large TPU
  • Medium TPU
  • Small TPU
  • Small NinjaFlex

#### 2.3.1 Antagonistic Actuators

The two types of antagonistic actuators used in this work are first, bellows continuum actuators, and second, Rotary Elastic Chamber actuators, shown in Figure 1.

##### 2.3.1.1 Bellows Continuum Actuators

The bellows continuum actuators provide a force from each pneumatic chamber that is related to the pressure in the bellows, resulting in a torque about the bottom plate of the actuator. Each joint in the case of our experiments has four independently controlled bellows. As the torques of all four actuators are summed a resultant torque causes movement in either (or both) of the two DOF. Both of the bellows continuum actuators we use in this paper are built with four actuators arranged in a square pattern such that the cross-section of the four actuator chambers form a square or diamond.

The continuum actuator in Figure 1A is made from blow-molded plastic bellows and identified in the rest of the paper as the **Blow-molded Continuum actuator**. The blow-molded bellows have a high stiffness and therefore a high return force to their neutral configuration.

The other continuum actuator in Figure 1B is made of rubberized fabric that was heat welded into bellows and is identified in the rest of the paper as the **Bead Continuum actuator**. The fabric from which this actuator is made has very little stiffness and therefore when this actuator is not pressurized it has negligible stiffness and collapses to its joint limits under its own weight.

Although the continuum actuators are two DOF actuators, we will only report the range of motion for a single bending plane. This is reasonable since the continuum actuators used in this work have an approximately identical range of motion for any plane in which they are bent.

##### 2.3.1.2 Rotary Elastic Chamber Actuators

The Rotary Elastic Chamber actuators (as described in Section 1.1) used in this work behave and rotate similar to a traditional pin joint actuator. The Rotary Elastic Chamber actuator in Figure 1C is made of the same rubberized fabric as the bead continuum actuator but is heat welded into bellows. It is identified in this paper as the **Rubberized Rotary Elastic Chamber actuator**. This actuator, similar to the Bead Continuum actuator has no stiffness and when it is not pressurized it moves to its joint limits under its own weight.

The last Rotary Elastic Chamber actuator, see Figure 1D, is built entirely out of fabric with internal bladders which, as they expand in the fabric, cause rotation about a fabric center of rotation. It is identified in the paper as the **Fabric Rotary Elastic Chamber actuator**.

Each of the antagonistic actuators in this work was designed and built by Otherlab Inc.

#### 2.3.2 Non-Antagonistic Actuators, Fluidic Elastic Actuators

**FIGURE 2:**
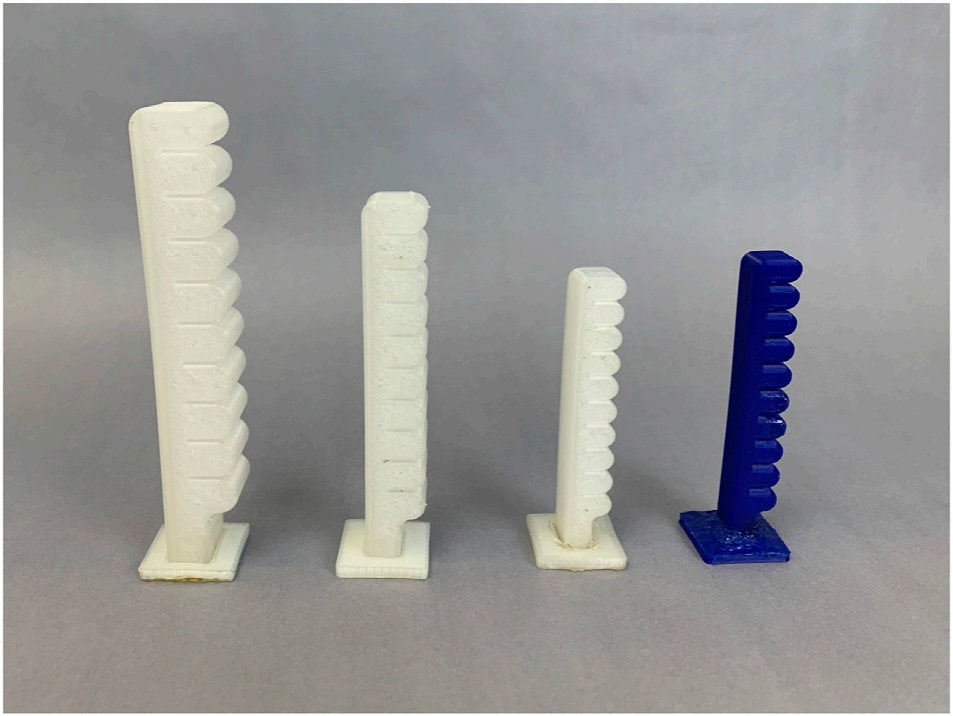
Fluidic Elastic Actuators used in this work from left to right: Large TPU, Medium TPU, Small TPU, and Small NinjaFlex.

The non-antagonistic actuators used in this work are Fluidic Elastic Actuators and are shown in Figure 2. They have simple pneumatic chambers with geometry that allows them to bend in a single direction as the pressure in the chamber increases. We built three different sizes (differentiated in the paper by small, medium, and large) as well as one size in two different materials using 3D printing techniques. The Medium Fluidic Elastic Actuator is 1.25*X* larger than the Small Fluidic Elastic Actuator in all dimensions while the Large Fluidic Elastic Actuator is 1.5*X* larger than the Small Fluidic Elastic Actuator. The two different materials are a clear TPU which has a Shore Hardness of 95A (SainSmart, 2019) and the other is NinjaFlex which has a Shore Hardness of 85A (Ninjatek, 2019).

While we explore only two dimensions of the design space, (i.e., size and material), many others could have been chosen. However, as stated previously, the purpose of this paper is to develop general metrics that can be used to evaluate actuators across many dimensions of the design space. As shown in Section 3 our limited design variations are able to show that the proposed metrics can enable effective Design Comparison.

In Table 1 we have included some dimensions of the different actuators to provide a sense of scale.

**TABLE 1:** Dimensions of each actuator.

	Bounding box l × w × h (m)	Maximum pressure kPa	Mass kg
Fabric rotary elastic chamber	0.191 × 0.191 × 0.305	102.0	1.52
Rubberized rotary elastic chamber	0.191 × 0.191 × 0.102	104.6	1.28
Blow-molded continuum	0.191 × 0.191 × 0.206	450.0	1.92
Bead continuum	0.229 × 0.229 × 0.382	171.6	8.44
Large TPU fluidic elastic actuator	0.0244 × 0.0244 × 0.172	238.2	0.0736
Medium TPU fluidic elastic actuator	0.0204 × 0.0204 × 0.143	244.6	0.0446
Small TPU fluidic elastic actuator	0.0163 × 0.0163 × 0.114	248.9	0.0251
Small NinjaFlex fluidic elastic actuator	0.0164 × 0.0164 × 0.119	243.5	0.0377

The dimensions of the bounding box includes that part of the actuators that is outlined in red in Figure 1.

### 2.4 Case Study

To demonstrate how these metrics can be used to evaluate actuators for a task we have included an example case study. Using a given task we analyze the metrics to evaluate which actuators would be of most value for that task.

In 2017 we took a survey of the soft robotics academic community using the robotics-worldwide mailing list (2021) and received 20 anonymous responses. Instead of fabricating a scenario for the example case study we instead use this survey as the basis for the case study. This survey asked the soft robotics community to rank the importance of several metrics as they related to five different tasks. For the case study we focus on the results of survey for a “Wiping Task” which was defined as “A task where a robot wipes a surface, whether an end effector or other parts of the manipulator are used. An example includes the cleaning of a solar panel.”

The survey participants ranked the importance of a metric as it pertained to a task as either “Extremely Important,” “Very Important,” “Moderately Important,” “Slightly Important” or “Not at all Important.” As the focus of this paper is developing actuator metrics the full results of the survey are not included but the full survey data can be found here https://bit.ly/38xe0fn.

To determine what metrics the community felt were most important, we used a weighted scoring method which is commonly used in engineering design process. The weighting was calculated as follows. For each individual who considered the metric as “Extremely Important”, the metric was given 4 points, for “Very Important” it was given 3 points, for “Moderately Important” 2 points, for “Slightly Important” 1 point, and 0 points for “Not at all Important.” The average of the points (the sum of the total points divided by the number of responses) was then used as the final score for the metric.

In Section 3.9, we present the results of the survey for the Wiping task and the analysis performed in the case study. Although the survey metrics are designed for a full robot manipulator, with some assumptions, most of them can be related to the actuator metrics proposed in this paper. By using these relations we are able to evaluate the suitability of the actuators presented in this paper based on the requirements defined for the wiping task.

## 3 Results

In the following sections (3.1 through 3.7) we present an analysis of the data we collected from different soft robot actuators along with an evaluation of each metric using the Metric Evaluation Criteria (MEC) discussed in the previous section.

As examples of the ability of our metrics to enable Design Comparison we present specific comparisons between similar actuators of different sizes as well as similar actuators made of different materials.

Additionally we use the rest of our results to present the importance of each metric with respect to the MECs for Task Utility and Information. Again the scope of this work is not to quantify precisely how these specific actuators perform, but to develop metrics by which soft fluid-driven actuators can be compared. Thus enabling a standardized method for discussing soft fluid-driven robot actuator performance. Table 2 summarizes the results of all the experiments for each actuator with respect to the different metrics.

**TABLE 2:** Summary of all metrics.

Actuator	Mass kg	Max Torque Nm	Torque-to-Mass Ratio Nm/kg	Efficiency Nm/J	Parasitic Stiffness Nm/rad	Variable Stiffness Nm/(rad*J)	Max range of motion rad
Fabric rotary elastic chamber	1.52	22.51	14.81	106.56	0.99	215.00	(−1.57, 1.57)
Rubberized rotary elastic chamber	1.28	47.26	36.92	254.21	N/A	183.50	(−1.31, 1.31)
Blow-molded continuum	1.92	63.63	33.14	266.66	21.26	16.81	(−1.41, 1.41)
Bead continuum	8.44	51.94	6.15	229.20	N/A	156.37	(−1.57, 1.57)
Large TPU fluidic elastic actuator	0.0736	2.43	33.06	119.10	6.65	N/A	(0, 1.88)
Medium TPU fluidic elastic actuator	0.0446	1.59	35.75	130.65	2.58	N/A	(0, 1.73)
Small TPU fluidic elastic actuator	0.0251	0.98	39.16	160.34	1.46	N/A	(0, 1.60)
Small NinjaFlex fluidic elastic actuator	0.0377	0.58	15.43	80.55	0.65	N/A	(0, 2.03)

**FIGURE 3:**
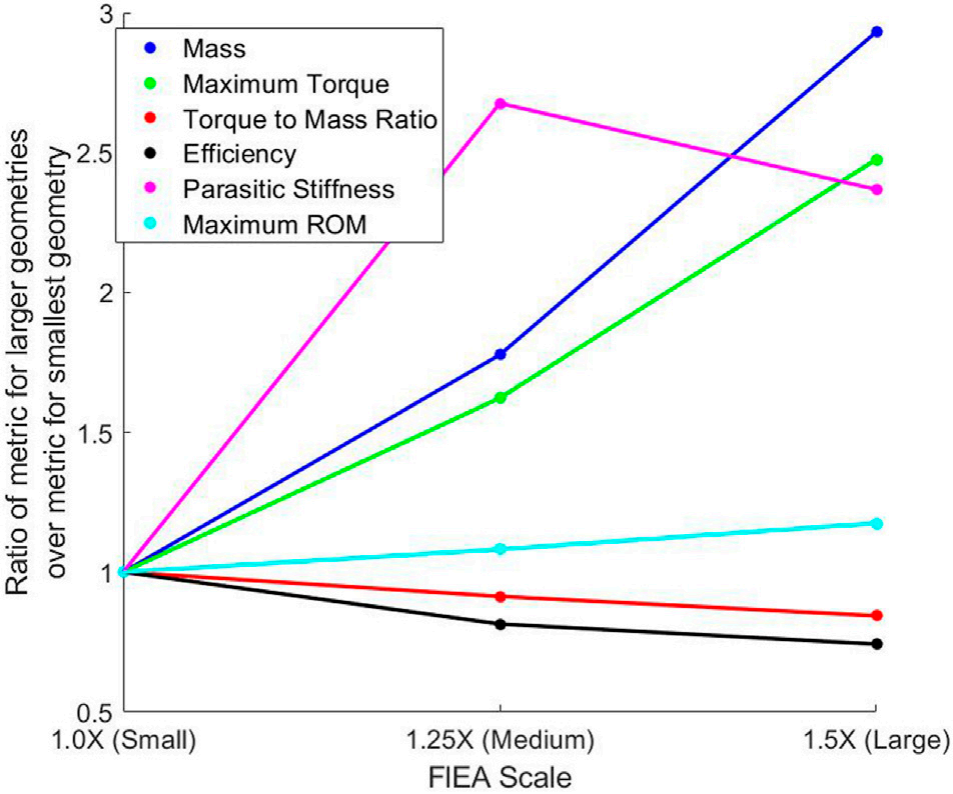
Plot of the metric values for the scaled TPU Fluidic Elastic Actuators divided by the metric values of the Small TPU Fluidic Elastic Actuator. The non-linear nature of most of the metrics means that there is not a one to one mapping between scaling and what the resultant metric will be.

Additionally, for making comparisons between the TPU Fluidic Elastic Actuator actuators for the metrics and their performance based on a variation in their scale, we have plotted the ratios of the different TPU Fluidic Elastic Actuators in Figure 3. This is done by dividing the values for each metric for the Small, Medium and Large TPU Fluidic Elastic Actuators by the value for each metric of the Small TPU Fluidic Elastic Actuators. We plot all the metrics in the same plot as a ratio so that a more effective visual comparison can be performed. By analyzing the varying slopes shown on Figure 3, a gross relationship between variation in actuator scale and each metric can be determined.

### 3.1 Mass

Although the mass of the actuators is not one of our proposed metrics, we use it as a means of comparison with the other metrics we are recommending.

#### 3.1.1 Design Comparison

When comparing the mass of the Fluidic Elastic Actuators as shown in Table 2, as expected, the scaled versions of the TPU actuators are larger and scale approximately linearly with the geometry. The NinjaFlex Fluidic Elastic Actuator has a similar size to the Small TPU Fluidic Elastic Actuator but has 50*%* more mass.

From Figure 3 it can be seen that as the scale increases the mass increases but at a higher rate. This can be seen by the steeper slope from Medium to Large Fluidic Elastic Actuator as compared to going from the Small to Medium.

The Rotary Elastic Chamber actuators have similar masses so no significant comparison can be made. While the continuum joints do have significantly different masses, their method of construction do not allow for good comparison using the Mass Metric. Much of the difference in mass is due to the top and bottom cap of the Bead Continuum actuator and not a variance in design.

The differences found in the slope of the Fluidic Elastic Actuators show that this metric does allow for comparison and Design Comparison MEC and is evaluated as “Yes” for the mass metric.

#### 3.1.2 Task Utility

The mass metric provides relevant data that can be used to guide selection for different applications. Therefore it is useful in terms of Task Utility as the mass of the robot is often an important factor for many soft robot applications therefore the Task Utility MEC is evaluated as “Yes” for the mass metric.

#### 3.1.3 Information

The Information MEC compares each metric to the other metrics to examine if the metric in question provides any new information. Both the mass metric and the Torque-to-Mass Ratio metric provide similar information about mass. So for the Information MEC only one can be used to satisfy the MEC. The authors feel that the Torque-to-Mass Ratio metric is a better candidate and evaluate the Information MEC for the mass metric as “No.” A more detailed discussion of this reasoning is found in Section 3.3.

Although this metric does have its use, it does not satisfy all three MEC and we therefore do not recommend it as a metric.

**Table:** 

Mass	
Design Comparison	Yes
Task Utility	Yes
Information	No

### 3.2 Maximum Torque

Of the antagonistic actuators the Blow-molded Continuum actuator has the ability to produce the largest maximum torque of 63.90 Nm at a pressure of 450.0 kPa as per the manufacturer’s specifications. The Fabric Rotary Elastic Chamber has the lowest maximum torque of 22.51 Nm at 102.0 kPa as per the manufacturer’s specifications. The maximum torque from the Fluidic Elastic Actuators was significantly smaller but this is expected from the relative size of the actuators.

#### 3.2.1 Design Comparison

Increasing the scale of the Fluidic Elastic Actuators did increase the maximum torque and as seen in Figure 3 where there is only a slight inflection as the scale keeps increasing. This can be seen by the almost constant slope for the Maximum Torque Metric in Figure 3.

The Small TPU Fluidic Elastic Actuator had a 1.69 times higher Max Torque than the Small NinjaFlex Fluidic Elastic Actuator. This shows that varying material can have a significant effect on the performance of an actuator with respect to this metric. A similar trend can be seen for the Rotary Elastic Chamber actuators. There is a significant increase in Maximum Torque from the Rubberized Rotary Elastic Chamber as compared to the Fabric Rotary Elastic Chamber. This demonstrates that this metric satisfies the Design Comparison MEC and is evaluated as “Yes.”

#### 3.2.2 Task Utility

Because many applications or tasks for which these actuators would be used have a payload requirement this metric is also important in terms of the MEC of Task Utility. We evaluate the Task Utility MEC as “Yes” for the Maximum Torque metric.

#### 3.2.3 Information

Lastly when comparing the other proposed metrics, this metric does share information with the Torque-to-Mass Ratio metric. As discussed previously we feel the Torque-to-Mass Ratio metric better satisfies the Information MEC with respect to mass. Therefore the Max Torque metric provides new information about the Maximum Torque of each actuator that is not readily available from the Torque-to-Mass Ratio exclusively. Therefore the Maximum Torque metric satisfies the Information MEC and is evaluated as “Yes”

**Table:** 

Maximum torque	
Design Comparison	Yes
Task Utility	Yes
Information	Yes

### 3.3 Torque-To-Mass Ratio

**FIGURE 4:**
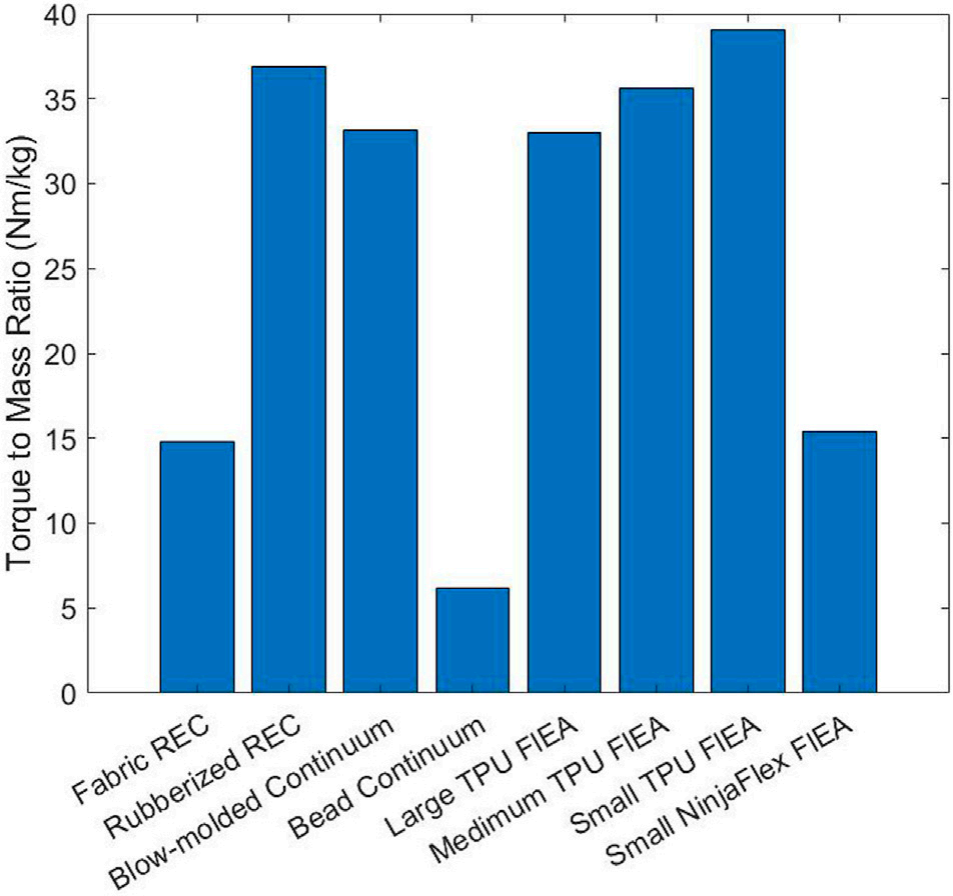
Bar plot of the Torque-to-Mass Ratios.

The joint with the greatest Torque-to-Mass Ratio is the Small TPU Fluidic Elastic Actuator with the Rubberized Rotary Elastic Chamber being the next highest with a close third of the Medium TPU Fluidic Elastic Actuators (see Figure 4). The obvious lowest is the Bead Continuum actuator, and although it has a high Max Torque, its large mass severely affects its Torque-to-Mass Ratio.

#### 3.3.1 Design Comparison

It can be noted that as the actuators were scaled to be larger the Torque-to-Mass Ratio decreased. As is shown in Figure 3, an increase in scale from the Small to Medium and from Medium to Large results in a decrease in the Torque-to-Mass Ratio. This comparison shows how this metric satisfies the Design Comparison MEC and is therefore evaluated as “Yes”

#### 3.3.2 Task Utility

Many applications are sensitive to the mass of the actuators used. This metric will help guide the selection of an actuator that will have the required payload while also limiting the mass of the actuator. We evaluate the Task Utility MEC as “Yes” for the Torque-to-Mass Ratio metric.

#### 3.3.3 Information

The mass, Maximum Torque and Torque-to-Mass Ratio metrics have somewhat redundant information relating to the torque output and mass of the actuators. However, to satisfy the Information MEC each metric must have new information that other metrics do not. Therefore only two of the three metrics can satisfy the Information MEC as there are two independent variables. We chose Maximum Torque and Torque-to-Mass Ratio because they are stronger candidates with respect to the other MECs. Both Maximum Torque and Torque-to-Mass Ratio can be used better for Design Comparison and Task Utility. We therefore evaluate the Information MEC as “Yes” for the Torque-to-Mass Ratio.

**Table:** 

Torque-to-Mass ratio	
Design Comparison	Yes
Task Utility	Yes
Information	Yes

### 3.4 Efficiency

**FIGURE 5:**
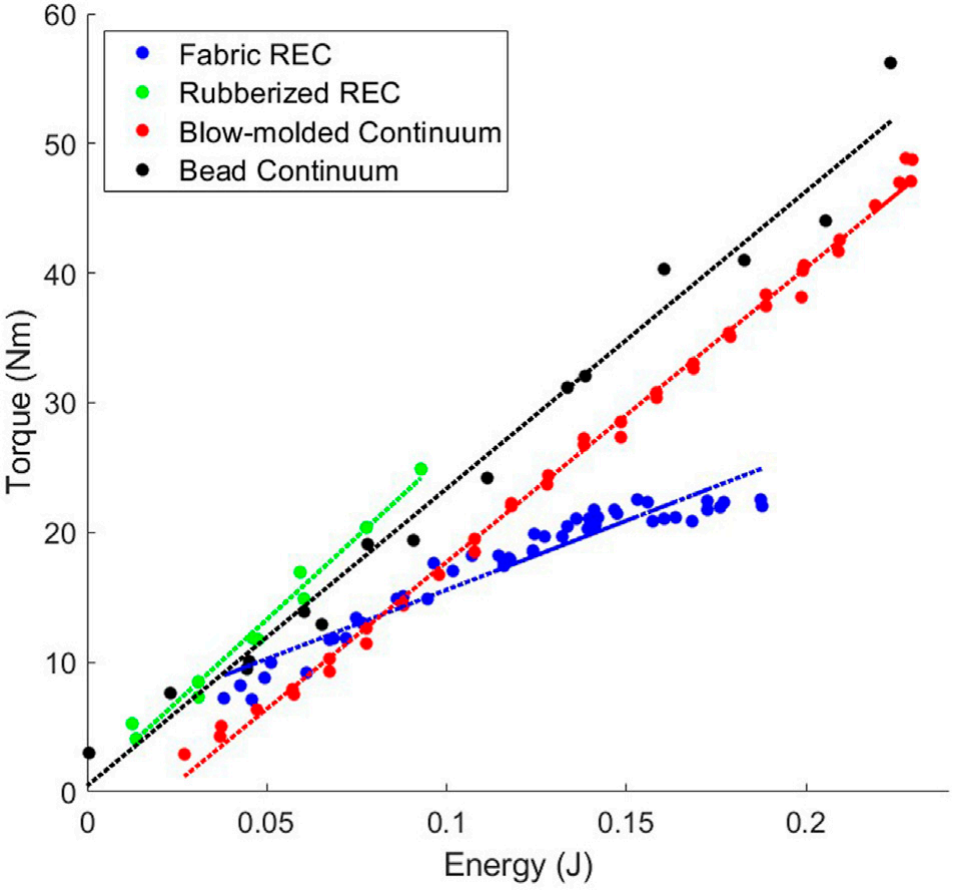
Relationship between energy and torque for the antagonistic actuators.

**FIGURE 6:**
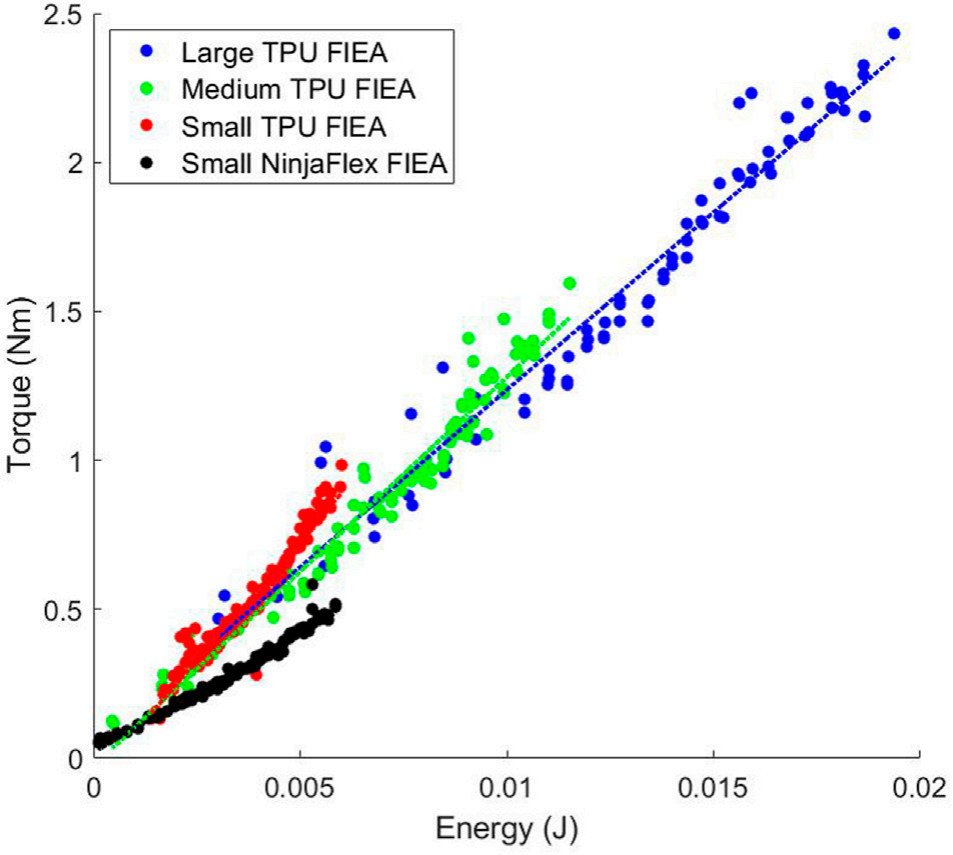
Relationship between energy and torque for the fluidic elastic actuators (FlEA). As the size of the actuator increases the amount of efficiency of the actuator decreased.

Figures 5, 6 show the efficiencies of all the actuators that we tested. As stated previously, we measure efficiency as the change in energy in the system as compared to the change in torque output. This correlatives to the slopes of the data found in Figures 5, 6. Because the antagonistic actuators can accept much higher input pressures (therefore containing more energy) than the Fluidic Elastic Actuators they were separated into two plots for readability.

As previously stated, the antagonistic actuators have much higher energies than the Fluidic Elastic Actuators. However, this does not mean that they are always more efficient as is shown in Table 2. The actuator with the largest efficiency that we tested is the Rubberized Rotary Elastic Chamber. Interestingly the two Continuum actuators, the Bead Continuum and Blow-molded Continuum actuators had very similar efficiencies. Of additional note is that three of the Fluidic Elastic Actuators, the Large, Medium and Small TPU Fluidic Elastic Actuators had higher efficiencies than the Fabric Rotary Elastic Chamber actuator.

#### 3.4.1 Design Comparison

As the Fluidic Elastic Actuators were scaled up the Efficiency did not increase, but in fact decreased as seen in Figure 3. However, it does seem that there is a slight change in slope as the Fluidic Elastic Actuator was scaled from Medium to Large as compared to scaling from Small to Medium. This suggests that the efficiency may start to increase again as the size continues to increase. Additionally, the Small TPU Fluidic Elastic Actuator is essentially two times more efficient than the Small NinjaFlex Fluidic Elastic Actuator. This kind of comparison shows the importance of evaluating the type of material used for soft robot actuators and how the metrics presented enable this comparison.

As seen in Figure 5 the efficiencies for the antagonistic actuators are similar with the exception of the Fabric Rotary Elastic Chamber. The efficiency of the Fabric Rotary Elastic Chamber (REC) is less than half the efficiency of the other antagonistic actuators.

As can be seen by these comparisons, this metric satisfies the Design Comparison MEC and is evaluated as “Yes.”

#### 3.4.2 Task Utility

Any application that has a limited supply of energy (or pressure) would consider the efficiency of an actuator when making a selection. Therefore this metric satisfies the Task Utility MEC and is evaluated as “Yes.”

#### 3.4.3 Information

This metric uses the energy which is unique from any of the other metrics discussed in this work. So the Information MEC is evaluated as “Yes” for this metric.

**Table:** 

Efficiency	
Design Comparison	Yes
Task Utility	Yes
Information	Yes

### 3.5 Parasitic Stiffness

As discussed in Section 2.3 the Rubberized Rotary Elastic Chamber and Bead Continuum actuators have practically no parasitic stiffness and will not be discussed in this section. The Blow-molded Continuum actuator had by far the largest parasitic stiffness while the other actuators’ ranges were similar.

#### 3.5.1 Design Comparison

For the Fluidic Elastic Actuators, as the size of the actuator increased the parasitic stiffness also increased from the Medium to the Small, but it decreased from the Medium to the Large Fluidic Elasctic Actuator. This variation in parasitic stiffness shows how not only will the selected material affect the stiffness of the actuator, but the geometry of the actuator will affect the stiffness as well.

Additionally, the difference in stiffness between the Fabric Rotary Elastic Chamber and the Blow-molded Continuum actuator is of significance. The trade-off between stiffer plastic and less stiff fabric is very apparent when comparing these two actuators with respect to Parasitic Stiffness. For this reason we evaluate the Design Comparison MEC as “Yes” for the Parasitic Stiffness metric.

#### 3.5.2 Task Utility

As the parasitic stiffness reduces the available torque when the actuators are bent, for many applications it is important to know how much torque is available over the desired range of motion. The parasitic stiffness metric will allow us to quantify if a soft robot actuator will have an acceptable stiffness for the range of motion of the task. Thus this metric satisfies the Task Utility MEC as is evaluated as “Yes.”

#### 3.5.3 Information

While the Variable Stiffness metric also measures stiffness, they measure the stiffness of a soft robot actuator from different sources. Therefore the unique information found in this metric shows that it satisfies the Information MEC and we evaluate it as “Yes.”

**Table:** 

Parasitic stiffness	
Design Comparison	Yes
Task Utility	Yes
Information	Yes

### 3.6 Variable Stiffness

**FIGURE 7:**
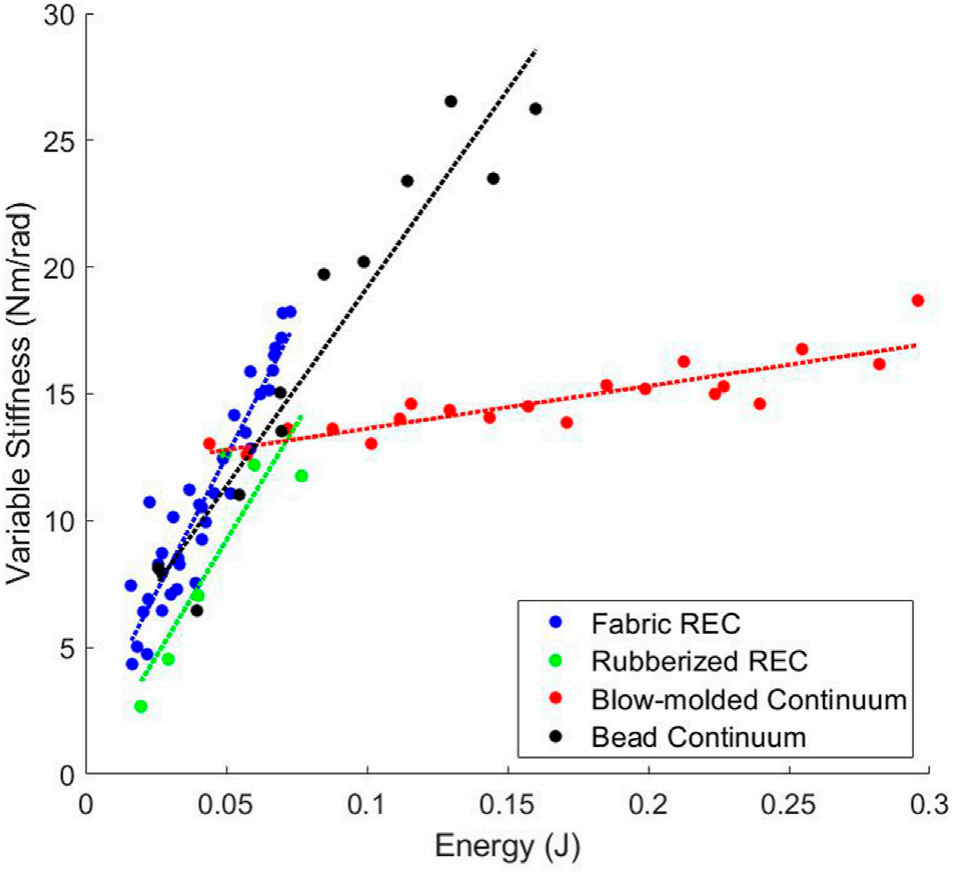
The variable stiffness relationship between pressure and stiffness for the antagonistic actuators.

As shown in Table 2 and Figure 7 the Fabric Rotary Elastic Chamber, Rubberized Rotary Elastic Chamber, and Bead Continuum actuators all have similar Variable Stiffness capabilities. However, the Blow-molded Continuum actuator is incapable of large changes in stiffness.

There is a significant difference in performance between the Blow-molded Continuum actuator and the other antagonistic actuators. The test on every actuator was performed in the exact same way and exploring this difference and its root cause is beyond the scope of this paper. However, the difference is clear.

#### 3.6.1 Design Comparison

By changing the material and design between the two Continuum actuators, there is a significant difference in potential variable stiffness. This means that simply because the actuators are antagonistic in nature, does not mean that they will allow significant changes in stiffness, even if other similar actuators do. Using this metric we are able to effectively compare the two continuum actuators showing that effective Design Comparison is enabled by this metric. Therefore we evaluate the Design Comparison MEC as “Yes” for this metric.

#### 3.6.2 Task Utility

For any application that requires a variable stiffness joint this metric is essential. Therefore we evaluate the Task Utility MEC as “Yes” for the Variable Stiffness metric.

#### 3.6.3 Information

As previously mentioned this metric does deal with stiffness like the previous metric, but it is a different type of stiffness that comes directly from actuator geometry (such as surface area) and applied pressures. Therefore this metric provides unique information and therefore satisfies the Information MEC and is evaluated as “Yes.”

**Table:** 

Variable stiffness	
Design Comparison	Yes
Task Utility	Yes
Information	Yes

### 3.7 Maximum Range of Motion

The range of motion of the antagonistic actuators were all very similar while the Fluidic Elastic Actuators had much more variance. The Fabric Rotary Elastic Chamber and Bead Continuum joints have the largest range of motion with (−1.57, 1.57) rad, while the Small TPU Fluidic Elastic Actuator has the smallest range of motion of (0, 1.60) rad.

#### 3.7.1 Design Comparison

As the Fluidic Elastic Actuators were scaled, the Maximum range of motion scaled at a constant rate as shown in Figure 3. Although this does allow for some Design Comparison the linear nature is somewhat trivial. However, when comparing the Fluidic Elastic Actuators, the Small NinjaFlex Fluidic Elastic Actuator (when inflated to its maximum operating pressure) had the highest range of motion with (0, 2.03) rad while the similarly sized Small TPU Fluidic Elastic Actuator had the lowest range of motion of (0, 1.60) rad. The difference in material had a much larger impact on the Maximum range of motion than the scaling and thus allows for a significant Design Comparison. Therefore this metric satisfies the Design Comparison MEC and we evaluate it as “Yes.”

#### 3.7.2 Task Utility

Because the range of motion of an actuator determines its workspace, it is key to understanding the range of motion when selecting an actuator for an application. The Task Utility MEC is evaluated as “Yes” for this metric.

#### 3.7.3 Information

As none of the other metrics can be used to determine the data in this metric, this metric satisfies the Information MEC and is evaluated as “Yes.”

**Table:** 

Variable stiffness	
Design Comparison	Yes
Task Utility	Yes
Information	Yes

### 3.8 Metric Evaluation

Table 3 summarizes the results of the metrics’ evaluations.

**TABLE 3:** Summary results from the metric evaluation using the MEC.

Evaluation category	Mass	Maximum torque	Torque-to-Mass ratio	Efficiency	Parasitic stiffness	Variable stiffness	Maximum range of motion
Design comparison	Yes	Yes	Yes	Yes	Yes	Yes	Yes
Task utility	Yes	Yes	Yes	Yes	Yes	Yes	Yes
Information	No	Yes	Yes	Yes	Yes	Yes	Yes

All of the proposed metrics, with the exception of mass, satisfied all three MECs. This shows that they are good metrics for soft robot actuators and can be used for making design comparisons, selecting actuators for different applications, and differentiating how different soft robot actuators perform.

### 3.9 Case Study

In order to show how the proposed metrics would be useful in selecting soft robot actuators for an actual task, or even potentially designing new actuators for a given task, we next present the metrics as applied to our case study from Section 2.4. The results of the analysis of the survey for the wiping task are included in Table 4. The seven metrics that were scored for the wiping task survey were:

1. Compliance at the End Effector (3.2 points)
2. The Hardness/Softness of the material making contact (3.05 points)
3. The ability to perform Force Control (3.05 points)
4. Compliance of the Joints and Links (2.9 points)
5. The Reachability of the serial manipulator (2.75 points)
6. The ability of the serial manipulator to maintain a Tolerance about a Trajectory (2.75 points)
7. The Time to Completion of the desired wiping task (2.75 points)

**TABLE 4:** Survey analysis results for the wiping task from a total of 20 responses.

	Compliance at EE	Force Control	Compliance	Tolerance about Trajectory	Reachablility	Timeto Completion	Hardness/Softness
Extremely Important (4 pts)	7	9	5	5	5	2	6
Very Important (3 pts)	10	4	9	7	7	6	10
Moderately Important (2 pts)	3	6	5	6	6	9	3
Slightly Important (1 pts)	0	1	1	2	2	3	1
Not at all Important (0 pts)	0	0	0	0	0	0	0
Average Importance Score	3.2	3.05	2.9	2.75	2.75	2.35	3.05
Standard Deviation	0.68	0.97	0.83	0.94	0.94	0.85	0.80

Each entry represents the tally of individuals that ranked the corresponding metric with the corresponding level of importance (e.g., Compliance at EE had seven individuals rank its importance at “Extremely Important” etc.). The average importance score is calculated by multiplying each row by the their respective row weightings (4 points, 3 points, etc.), summing the columns and dividing by the total number of samples (20).

As the two metrics Tolerance about Trajectory and Time to Completion are dynamic measures and depend heavily on the controller and dynamics of the system, we will not explore them in this case study. In order to relate the manipulator metrics from the survey to the actuator metrics some assumptions have been made and will be stated as necessary.

Both of the compliance metrics from the survey are related to the Variable Stiffness metric proposed in this paper as the compliance of a manipulator is directly tied to the compliance of the actuators (see Albu-Schäffer and Bicchi, 2016, Section 21.6). Because there is no compliance value or range specified we assume that a manipulator that has the ability to vary its compliance over the widest range will provide the most utility. By having a variable stiffness actuator the compliance can be adapted for varying scenarios of a wiping task. To satisfy this metric the Blow-molded Continuum joint can be ruled out since it has almost no ability to vary stiffness. However, the other three antagonistic actuators seem like possible candidates.

The Hardness/Softness metric from the survey does not directly correlate to any of the metrics proposed in this paper. However, the hardness of a object is related to its deflection properties by the modulus of elasticity, and as discussed in Section 3.5, the stiffness of the material from which the actuators are made has a correlation to the Parasitic Stiffness. We also assume that for a wiping task the hardness of the actuators should be minimized so that any impact forces can be reduced. Therefore, looking at the Parasitic Stiffness, the Blow-molded Continuum joint can be ruled out again due to its high Parasitic Stiffness and the Rubberized Rotary Elastic Chamber and Bead Continuum joint have rigid plastic elements that remove them as ideal candidates. Therefore the Fabric Rotary Elastic Chamber is the best choice out of the antagonistic actuators which can actually provide variable stiffness outputs.

The ability to have force control is related to the Maximum Torque metric proposed in this paper. The larger the maximum torque that an actuator can achieve, the wider the range of force control that is theoretically possible. While the resolution of the force control is also a factor to consider, for this analysis we are assuming that a wide range of force control is of higher value and the resolution of the force control is adequate. Future work could include metrics that deal with control resolution for soft robot actuators. Therefore, for the force control metric, the Rubberized Rotary Elastic Chamber may be the best choice based on the Maximum Torque metric.

The Reachability metric of the survey can be directly related to the range of motion metric proposed in this paper. Generally the larger the range of motion of the actuators the higher the Reachability of the full serial manipulator will be. The Fabric Rotary Elastic Chamber and Bead Continuum actuators have the highest range of motion of ±1.57*rad* and would be the best candidates based on this metric. However the Bead Continuum joint is a slightly better choice due to its two degrees of freedom bending. It is important to note here that in the case of applying these metrics to robot design (and not just selection of existing actuators), there can be interaction between the metrics. For example, having a large range of motion may also require long soft robot links to achieve good reachability. Having good reachability does not mean that the designed manipulator would be able to lift the actual load of the soft robot designed. This then ties back to the Maximum Torque metric and we can see how the desired value for that may need to increase if the weight of the manipulator increases. However, the proposed metrics so far cover this use-case completely and would allow that trade-off in the design space if properly modeled.

As with many design decisions the engineer must make a choice on how to weight the importance of each metric. Based on the weighting we calculated from the survey, and if selecting only from the actuators presented in this paper, we would choose the Fabric Rotary Elastic Chamber for a serial manipulator used to perform wiping motions while in contact. These choices satisfy the Compliance metric, the Hardness/Softness metric and the Reachability metric. The design trade-off in this case requires sacrificing the actuator’s ability to perform Force Control in favor of the other metrics.

It should be noted that these may not be the “optimal” actuators for this task as the comparison in this paper is limited to only eight actuators. However, by using these metrics with accurate models during the actuator design phase, an “optimal” actuator can be designed and selected for a desired task. Although it did not include actuator design, this type of design optimization was shown for the whole kinematic structure of a soft robot manipulator in Bodily et al. (2017).

For this case study we are not proposing that the survey, the scoring method used, or our assumptions are the only or best methods for determining which metrics should be used to evaluate actuators for a task. As the task is more clearly defined, or if any of the assumptions change, then the final analysis and actuator choice would also change. Instead, we are providing an illustrative example of how the metrics can be used to select appropriate actuators for a given task.

## 4 Discussion

This paper is a step towards developing a unifying method to compare and evaluate soft robot actuators. This is necessary since there is currently no standard in the literature to enable comparison between soft robot actuators. We accomplish this by first developing a method of evaluating potential metrics using the Metric Evaluation Criteria which include Design Comparison, Task Utility, and Information. By using the MECs we were able to show that the metrics proposed in this work are effective metrics for fluidic soft robot rotational actuators. In our case study we also demonstrated how the metrics could be used to evaluate the actuators in this paper for the given task, thus showing how these metrics facilitate design and comparison of new and existing soft robot actuators within an engineering methodology.

It is important to note that while we have demonstrated the efficacy of these metrics for fluidic rotational actuators these metrics can be modified/adapted for use with other types soft robot actuators.

For non-fluidic rotational soft robot actuators only the Efficiency and Variable Stiffness metric will need significant modification. For both metrics it will be necessary to develop a method for measuring the potential energy of the system and the torque output during a static loading scenario. However once this has been done, the metrics in this paper are applicable for any other type of rotational soft robot actuator.

The metrics can also be adapted for soft robotic actuators that do not have rotational motion profiles. For linear actuators the metrics can be modified by replacing all torque and rotational measurements with force and linear measurements respectively. We readily acknowledge that the metrics described in this paper may not be adaptable to all soft robot actuators. As the motion profiles of the soft robot actuators become less general the metrics that need to be used to evaluate those actuators will also become less general. Examples of these actuators include twisting actuators and actuators that use eversion (Hawkes et al., 2017) as their actuation method. As more actuation methods (i.e., fluidic, electro static, etc.) and actuation modes (i.e., rotational, linear, twisting, etc.) are developed it will be important to develop metrics to start comparing them and characterizing their performance. What we have demonstrated in this paper is an important step in that effort.

### 4.1 Conclusion

Future work includes using the metrics developed here to evaluate additional soft robot actuators to enable the soft robotics community to make more rigorous comparisons between different soft robot actuation methods and designs. Some additional metrics that we feel should be explored include reliable life cycle, motion repeatablility, and safety to name a few. As the metrics in this paper are limited to static metrics, in future work it will also be important to develop dynamic metrics (related to actuator bandwidth and control bandwidth for soft actuators). Additionally the metrics presented can be expanded to encompass all soft robot actuators and not only fluidic rotational actuators as described above. We further expect that new potential soft robot applications will emphasize and clarify additional necessary requirements for comparison and evaluation of potential actuators.

Therefore, despite having developed six soft robot actuation metrics as well as guidelines for evaluation, we fully expect this set of guidelines to further mature as the field of soft robotics continues to grow.

## Data Availability

The raw data supporting the conclusions of this article will be made available by the authors, without undue reservation.
